# Identification of hub genes and pathways in Uterine corpus endometrial carcinoma (UCEC): A comprehensive *in silico* study

**DOI:** 10.1016/j.bbrep.2024.101860

**Published:** 2024-11-01

**Authors:** Mahsa Ejlalidiz, Ameneh Mehri-Ghahfarrokhi, Mohammadreza Saberiyan

**Affiliations:** a Medical Student Research Committee, School of Medicine, Shahid Beheshti University of Medical Sciences, Tehran, Iran; b Clinical Research Developmental Unit, Hajar Hospital, Shahrekord University of Medical Sciences, Shahrekord, Iran; c Department of Medical Genetics, School of Medical Sciences, Hormozgan University of Medical Sciences, Bandar Abbas, Iran

**Keywords:** Endometriosis, UCEC, PPI network, Diagnostic biomarkers

## Abstract

**Background:**

Uterine corpus endometrial carcinoma (UCEC), derived from the endometrium, is the most common type of endometrial malignasis. This gynecological malignancy is very common all over the world, especially in developed countries and shows a potentially rising trend correlated with the increase in obese women.

**Methods:**

Differentially Expressed Genes (DEGs) analysis was conducted on GSE7305 and GSE25628 datasets from the Gene Expression Omnibus (GEO). DEGs were identified using GEO2R (adjusted p-value <0.05, |logFC| > 1). Pathway analysis employed KEGG and Gene Ontology databases, while protein-protein interactions were analyzed using Cytoscape and Gephi. GEPIA was used for target gene validation.

**Results:**

We have identified 304 common DEGs and 78 hub genes using GEO and PPI analysis, respectively. The GO and KEGG pathways analysis revealed enrichment of DEGs in extracellular matrix structural constituent, extracellular space, cell adhesion, and ECM-receptor interaction. GEPIA analysis identified three genes, ENG, GNG4, and ECT2, whose expression significantly differed between normal and tumor samples.

**Conclusion:**

This analysis study identified the hub genes and associated pathways involved in the pathogenesis of UCEC. The identified hub genes exhibit remarkable potential as diagnostic biomarkers, providing a significant opportunity for early diagnosis and more effective therapeutic approaches for UCEC.

## Highlights

• Common DEGs and 78 hub genes were identified using GEO and PPI analysis, respectively.
• The GO and KEGG pathways analysis revealed enrichment of DEGs in cell adhesion, extracellular space, extracellular matrix structural constituent, and ECM-receptor interaction.
• The expression of ENG, GNG4 and ECT2 showed a significant difference between normal and tumor samples, have been identified by GEPIA analysis.

## 1 Introduction

Uterine corpus endometrial carcinoma (UCEC), a malignancy that develops in the lining of the endometrium, is the most prevalent type of endometrial cancer. This gynecological malignancy is very common all over the world, especially in developed countries and appears to be potentially rising in correlation with the growing prevalence of obesity among women. The UCEC's prevalence escalates with advancing age, particularly among women within the ages of 45 and 65 are affected by this disease [[1], [2], [3], [4], [5]]. Among the four subtypes of UCEC including serous or clear cell, endometrioid, endometrial carcinoma is the main histological type [6,7]. Moreover, UCEC is one of the primary contributors to mortality associated with malignancies among women in worldwide. The International Agency for Research on Cancer reports the UCEC's global incidence: 8.7 per 100,000 women and age-standardized death rates (ASRs) for this disease is 1.8 per 100,000 women [8]. UCEC has genetic heterogeneity and complex etiology, which is why its molecular basis is still not fully understood. Numerous endeavors have been undertaken to comprehend the pathophysiology of the disease, but there is still no good prognosis, especially for advanced UCEC [9]. Although the prognosis in women with UCEC is relatively good, recurrence is more likely in those with high-risk disease [10]. In fact, both the increase in incidence and mortality have established UCEC as a significant concern in women's health.

Although progress has been achieved in the early diagnosis of UCEC, and novel emerge treatments, the prognosis for advanced UCEC is not satisfactory, and a large number of women with more aggressive types of the disease have poor clinical outcomes [11,12]. Previous studies have sought to identify multiple UCEC biomarkers, including heat shock protein family A member 5 [13], activated leukocyte cell adhesion molecule [14], L1 cell adhesion molecule [15], sperm-associated antigen 9 [16], CD151 molecule (Raph blood group) [17], and progestogen-associated endometrial protein [13]. In this regard, the current study intended to identify hub genes and associated pathways involved in the UCEC's development to expand our understanding of the disease and seek potential biomarkers. We downloaded two datasets (GSE7305 and from the Gene Expression Omnibus (GEO, http://www.ncbi.nlm.nih.gov/geo/) to identify common differentially expressed genes (DEGs) using the GEO2R tool. Following the identification of pathways utilizing Gene Ontology (GO) and Kyoto Encyclopedia of Genes and Genomes (KEGG) databases, the Cytoscap and Gephi were applied to protein-protein interactions (PPIs) analysis. Then, the target genes were confirmed by Gene Expression Profiling Interactive Analysis (GEPIA) analysis. Collectively, our results offer novel perspectives into enhanced comprehension of the mechanisms underlying the development and progression of UCEC will enable us to plan effective treatments. Following a good prognosis and analyzing the expression of genes and pathways related to them, timely diagnosis of the disease will be provided and will lead to a reduction in mortality and optimization of treatment strategies.

## 2 Methods and materials

### 2.1 Microarray data

The Gene Expression Omnibus (GEO) repository is an essential resource for academics, providing open access to a comprehensive collection of microarray gene expression datasets. This extensive database has proven essential for conducting large-scale genomic research across several biological fields. We utilized a stringent selection approach to locate pertinent datasets that correspond with our study aims. The factors for our pick were as follows: 1) Human endometriosis specimens: The datasets must have gene expression profiles obtained from human endometrial tissue impacted by endometriosis. 2) Case-control research design: For comparison analysis, the chosen datasets must have both sick (case) and healthy (control) tissue samples. 3) Adequate sample size: A minimum of 20 samples was necessary to establish statistical robustness and reduce possible biases linked to small sample sizes.

Two datasets were discovered and obtained from the GEO database (http://www.ncbi.nlm.nih.gov/geo/) based on these rigorous criteria: GSE7305: This dataset consists of 20 human tissue samples, evenly divided between diseased endometrial (n = 10) and normal endometrium (n = 10). The dataset employed the Affymetrix Human Genome U133 Plus 2.0 Array (GPL570) microarray platform. GSE25628: This dataset comprises 22 samples, including 16 ectopic endometrium specimens from patients with endometriosis and 6 normal endometrial tissue samples. The gene expression profiling for this dataset was performed with the Affymetrix Human Genome U133A 2.0 Array (GPL571). The selected datasets fulfill our established criteria and offer a varied representation of endometrial tissue types, encompassing both eutopic (GSE7305) and ectopic (GSE25628) endometrium. This variability facilitates a more thorough investigation of the molecular pathways involved in endometriosis etiology.

### 2.2 Common DEGs

The identification of shared differentially expressed genes (DEGs) in patient and control samples was performed using GEO2R (https://www.ncbi.nlm.nih.gov/geo/geo2r/), a web-based analytical tool offered by the Gene Expression Omnibus. To guarantee statistical significance and biological relevance, rigorous criteria were implemented for differentially expressed gene (DEG) selection: adjusted P-values <0.05, log2 fold change (FC) > 1 for upregulation, and log2 FC ≤ −1 for downregulation. These metrics were utilized to develop an extensive gene expression network. Venn diagrams were created with the Evolutionary Genomics Venn diagram tool (http://bioinformatics.psb.ugent.be/webtools/Venn/) to enable the comparison and display of DEGs across datasets. This method facilitated the precise distinction of overlapping and distinct DEGs between the upregulated and downregulated gene sets. The resultant Venn diagrams provided a basis for further bioinformatic analysis, facilitating a more refined comprehension of the transcriptome changes linked to endometriosis.

### 2.3 Functional enrichment analysis

A detailed functional enrichment analysis was conducted to clarify the biological implications of the discovered DEGs. This investigation included several aspects of cellular biology, such as cellular components (CC), molecular functions (MF), and biological processes (BP). The Gene Ontology (GO) database (http://www.geneontology.org) was employed for this purpose, since it is one of the most extensively utilized bioinformatics resources for large-scale functional annotation of genes and their products.

In addition to the GO analysis, the Kyoto Encyclopedia of Genes and Genomes (KEGG) database (https://www.kegg.jp/) was utilized to examine pathway enrichment. KEGG provides a comprehensive database of molecular information and aids in the analysis of high-throughput data inside biological systems. This method facilitated the discovery of signaling pathways and metabolic processes that may be dysregulated in endometriosis. The actual execution of these analyses was conducted utilizing the Database for Annotation, Visualization, and Integrated Discovery (DAVID) (https://david.ncifcrf.gov/). DAVID offers an extensive array of functional annotation tools for researchers to comprehend the biological significance of extensive gene lists. A threshold of P < 0.05 was imposed for all enrichment studies to indicate statistical significance, hence assuring the robustness of the discovered functional categories and pathways.

### 2.4 PPI network construction and performance analysis

The DEGs were uploaded to the STRING database (https://string-db.org; version 11.5) to create a PPI network. The network was studied to discover hub genes, which are fundamental to its structure and perhaps critical in the illness process. The PPI network data produced by STRING was imported into Cytoscape (version 3.6.0) for additional analysis and visualization.

Hub genes were found in Cytoscape using three principal centrality metrics: closeness, betweenness, and degree. These metrics assess many dimensions of a gene's significance within the network structure. The resultant network data, encompassing the identified hub genes, was subsequently exported from Cytoscape and loaded into Gephi software for enhanced visualization. In Gephi, the hub genes were graphically accentuated and categorized according to their network characteristics, offering insights into possible functional linkages among these pivotal molecular contributors to endometriosis pathogenesis.

### 2.5 Verification and survival analysis

Differential mRNA expression analysis was performed with GEPIA's “Single Gene Analysis" module to identify biomarkers associated with UCEC. An interactive web server named GEPIA has been developed by Peking University, which aims to analyze and visualize RNA-sequencing expression data.

### 2.6 Statistical analysis

Following the calculating the values of DEGs taken from GEO DataSets, and considering the significance threshold of P-value <0.05, GO enrichment analyses were conducted. In this way, the expression levels of UCEC related genes were examined using p-values <0.05, log2FC < 1, and matching TCGA Normal to GTEx data.

## 3 Results

### 3.1 Common DEGs

**Fig. 1:**
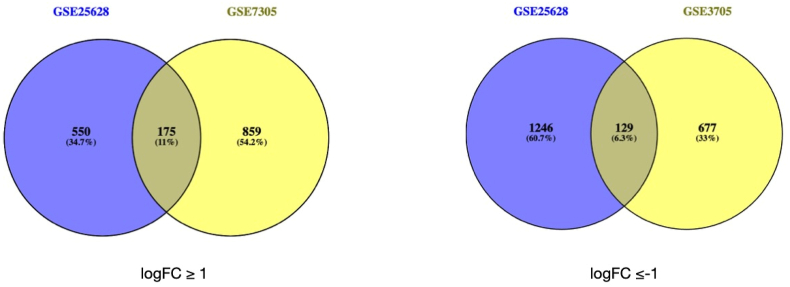
Identification of 304 common DEGs among GSE7305 and GSE25628 datasets by Venn diagram software. The Bioinformatics and Evolutionary Genomics Venn with a logFC ≥1 and logFC ≤ −1.

Two data sets including GSE7305 and GSE25628 were selected from the GEO database. Venn diagram software was applied for the data to determine common DEGs between the two data sets (Fig. 1). The results included 304 common DEGs, of which 175 upregulated and 129 downregulated DEGs (These results are provided in Supplementary Table 1).

### 3.2 GO and KEGG pathway enrichment of the common DEGs

Through DAVID database and Enrichr database, GO annotation and KEGG pathway enrichment analysis were performed respectively. Table 1 shows ten GO and KEGG enriched pathways. Table 1 illustrates that the GO biological process analysis indicated high enrichment of these 304 DEGs in cell adhesion, cell-cell adhesion, kidney development and negative regulation of membrane protein ectodomain proteolysis. Extracellular space, cell surface, basement membrane and extracellular exosome are 4 cellular components that are significantly enriched. The top 4 enriched terms of structural composition to analyze the molecular function of GO include extracellular matrix (ECM), integrin binding, beta-amyloid binding and protein binding, cell adhesion molecules, and complement and coagulation cascades.

**Table 1:** Key Pathways and Functions Associated with common DEGs in UCEC.

Category	Term	Count	P-Value
**GOTERM_BP_DIRECT**	GO:0007155∼cell adhesion	29	2.50047673243061E-09
	GO:0098609∼cell-cell adhesion	14	2.64417934956445E-06
	GO:0001822∼kidney development	11	5.48734211365512E-06
	GO:0051045∼negative regulation of membrane protein ectodomain proteolysis	4	1.34084356187903E-04
	GO:0009410∼response to xenobiotic stimulus	13	1.76766742340566E-04
	GO:1905821∼positive regulation of chromosome condensation	4	1.99096026233E-04
	GO:0070301∼cellular response to hydrogen peroxide	7	3.04681102931167E-04
	GO:0006958∼complement activation, classical pathway	6	3.21529176670062E-04
	GO:1905820∼positive regulation of chromosome separation	4	3.83232519690081E-04
	GO:0007179∼TGF-β receptor signaling pathway	8	4.44801551827947E-04
**GOTERM_CC_DIRECT**	GO:0005615∼extracellular space	58	5.86107640404059E-09
	GO:0009986∼cell surface	30	1.0171692546024E-08
	GO:0005604∼basement membrane	12	2.98877922127083E-08
	GO:0070062∼extracellular exosome	61	7.56643484002605E-08
	GO:0005576∼extracellular region	59	1.44986831200153E-07
	GO:0031012∼ECM	16	2.084009175593E-07
	GO:0005923∼bicellular tight junction	12	1.30844323237061E-06
	GO:0000796∼condensin complex	4	1.82364694518497E-04
	GO:0016323∼basolateral plasma membrane	13	2.01626271798142E-04
	GO:0005788∼endoplasmic reticulum lumen	14	2.70688421114552E-04
**GOTERM_MF_DIRECT**	GO:0005201∼ECM structural constituent	16	1.20195745984562E-10
	GO:0005178∼integrin binding	14	3.74883032599999E-07
	GO:0001540∼beta-amyloid binding	8	2.32896641310804E-04
	GO:0005515∼protein binding	206	4.0525758357468E-04
	GO:0005509∼calcium ion binding	23	9.74217058006634E-04
	GO:0003779∼actin binding	14	0.001161556
	GO:0030021∼ECM structural constituent conferring compression resistance	4	0.001365846
	GO:0030169∼low-density lipoprotein particle binding	4	0.001641286
	GO:0005518∼collagen binding	6	0.002252549
	GO:0042802∼identical protein binding	40	0.002508921
**KEGG_PATHWAY**	hsa04512:ECM-receptor interaction	12	4.34912468830043E-07
	hsa05165:Human papillomavirus infection	19	2.20129454422999E-05
	hsa04514:Cell adhesion molecules	11	4.72510022129723E-04
	hsa04610:Complement and coagulation cascades	8	8.12810102643477E-04
	hsa04510:Focal adhesion	12	9.63344216555539E-04
	hsa04151:PI3K-Akt signaling pathway	15	0.004668709
	hsa04145:Phagosome	9	0.005637821
	hsa04530:Tight junction	9	0.010791298
	hsa04350:TGF-beta signaling pathway	7	0.012397517
	hsa04670:Leukocyte transendothelial migration	7	0.016487686

### 3.3 PPI network and hub genes

**Fig. 2:**
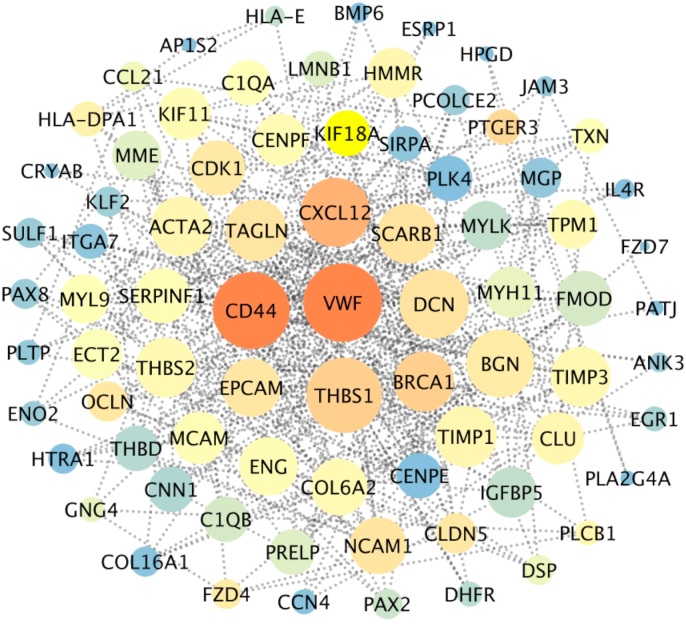
Comprehensive PPI network analysis of hub genes in UCEC: Visualization of key players and their interconnections derived from differential gene expression in GSE7305 and GSE25628 datasets.

By employing STRING database and Cytoscape software, a PPI network was drawn (Fig. 2). Analysis of PPI networks leads to the identification of strong molecular interactions that influence disease progression. The total number of nodes determined as DEG in the network was 234 (number of nodes: 234, clustering coefficient: 0.338, network centrality: 0.136). Hub genes were ranked based on degree, closeness, and betweenness centrality (Supplementary Table 2). To ascertain common genes in the top 78 genes as key hubs of the network (number of nodes: 78, clustering coefficient: 0.493, network centrality: 0.253) (Supplementary Table 3) STRING server and key hub identification parameters in the network were used.

### 3.4 Clustering of hub genes

**Fig. 3:**
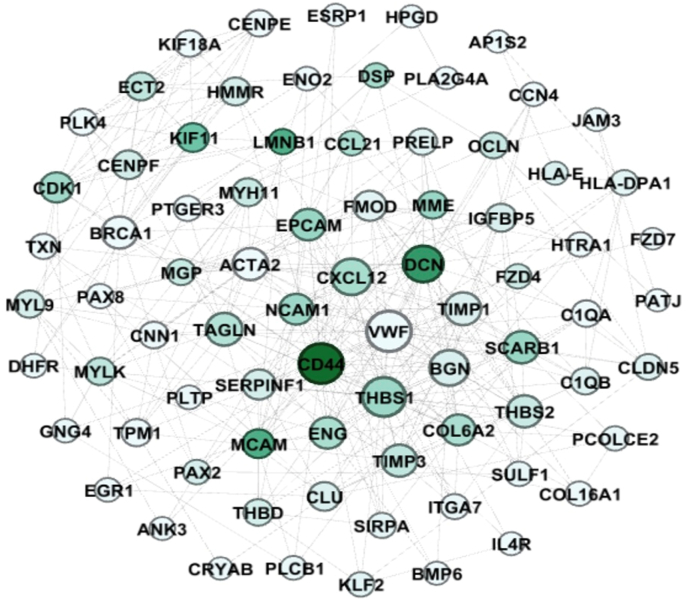
Visualization and analysis of UCEC's hub genes from the PPI network using Gephi. The dimensions and hue of nodes signify the degree and betweenness, respectively.

To visualize and analyze the interconnections among key genes, we employed Gephi version 0.9.2, a network analysis software. This tool facilitated the reconstruction of PPI network, specifically focusing on the identified hub genes (Fig. 3). A diagram of interaction between hub genes is shown in Table 2. Totally, 7 modules were determined as clusters in the network and then the centrality of each module was determined by calculating the centrality parameters for each module.

**Table 2:** The list of high-scored genes in UCEC calculated via Gephi software.

	Modules						
	1	2	3	4	5	6	7
**Gene symbols**	TAGLN	THBS1	CD44	BRCA1	CLU	VWF	PTGER3
	ACTA2	DCN	CXCL12	CDK1	C1QA	THBD	**GNG4**
	MYH11	BGN	SCARB1	CENPF	C1QB	OCLN	PLCB1
	MYL9	TIMP1	EPCAM	KIF11	HLA-E	CLDN5	
	TPM1	TIMP3	**ENG**	**ECT2**	HLA-DPA1	DSP	
	MYLK	THBS2	NCAM1	HMMR	CCL21	KLF2	
	CNN1	COL6A2	MCAM	CENPE	PLTP	IL4R	
	MGP	FMOD	MME	PLK4	EGR1	JAM3	
		SERPINF1	SIRPA	KIF18A	CRYAB	PATJ	
		IGFBP5	TXN	LMNB1	AP1S2		
		PRELP	PAX2	DHFR			
		ITGA7	PAX8				
		PCOLCE2	ENO2				
		COL16A1	ANK3				
		SULF1	BMP6				
		HTRA1	ESRP1				
		CCN4					

### 3.5 Verification of UCEC's hub genes

**Fig. 4:**
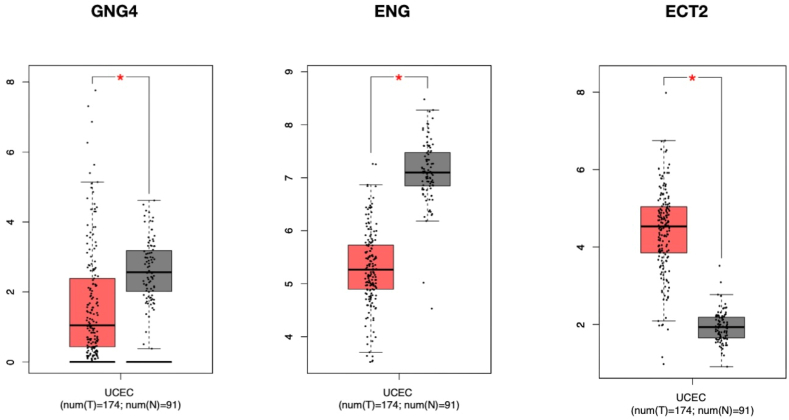
The UCEC's core genes presented in Box plots. GNG4, ENG and ECT2 revealed a substantial disparity between normal and malignant specimens (∗P < 0.05).

Significant prognostic value of some genes in UCEC has been shown by GEPIA. Some genes are significantly expressed between normal and tumor samples, so perhaps such genes can be introduced as potential biomarkers for UCEC (Fig. 4). In the present study, 3 genes whose expression was significantly different between normal samples and tumor samples were represented as potent biomarkers for UCEC (P < 0.05 for all genes). Among these genes, the overexpressed genes such as GNG4 in module 7 and endoglin (ENG) in module 3, as well as the decreased expressed genes such as epithelial cell transformation 2 (ECT2) in module 4 can be mentioned.

## 4 Discussion

Since UCEC is a poor prognostic (for aggressive types of the disease) gynecological malignancy with increasing incidence and high recurrence rate and mortality rate, understanding of the molecular mechanism of this, will have a substantial effect on the treatment and management policy. In other words, such studies can lead to the identification of appropriate biomarkers and approaches capable of forecasting disease prognosis [8,9].

In the present study, using bioinformatic evidence and reanalyzing published microarray data, we identified a total of 304 DEGs across two datasets (GSE7305 and GSE25628) obtained from endometrioses and normal specimens. These DEGs included 175 up and 129 downregulated genes in endometriosis patients. GO and KEGG analyses revealed that these DEGs are participated in cell adhesion, cell-cell adhesion, kidney development and negative regulation of membrane protein ectodomain proteolysis. The four components terms were significantly enriched include extracellular space, cell surface, basement membrane and extracellular exosome**.** GO molecular function analysis lead to determination of 4 significantly enriched terms include ECM structural constituent, integrin binding, beta-amyloid binding and protein binding. Finally, human papillomavirus infection, ECM-receptor interaction, complement and coagulation cascades, and cell adhesion molecules were the top four significantly enriched pathways for 304 UCEC's DEGs. Hub genes in various types of cancers like breast [18], colorectal [19], and non-small cell lung [20] cancers, have been obtained using DEGs and bioinformatics analysis of PPI. Today, bioinformatics analysis and data mining has helped research groups to identify disease prognostic biomarkers via relevant datasets ([18,21]). In recent years, approaches such as NGS and microarray have been used in the analysis of large-scale data related to the UCEC and the basic processes of the disease have been investigated [9,22].

In the present study three key genes in UCEC include Endoglin, GNG4 and epithelial cell transforming 2 (ECT2) identified, therefore enhancing our comprehension of UCEC and broadening insights into the prognosis for this cancer type. In previous studies on a variety of cancers, it has been seen that this gene participates in important facets of tumor development.

CD105, alternatively referred to as Endoglin (ENG), functions as an auxiliary type-III receptor within the TGF-β superfamily signaling cascade. This protein plays a crucial role in modulating angiogenesis and maintaining equilibrium in TGF-β signaling, thereby influencing the proliferation and movement of endothelial cells [23]. The TGF-β pathway exerts significant control over various cellular processes, including cell division, specialization, motility, and programmed cell death. ENG's involvement in these fundamental biological mechanisms underscores its potential importance in both typical physiology and pathological conditions [24]. Some previous investigations mentioned the impacts of endoglin on angiogenesis and tumor development [[25], [26], [27], [28]]. Tumor behavior can be directly influenced by the processes in which endoglin is involved. Studies have shown that ENG expression is not limited to tumor vessels and increased endoglin expression has been confirmed in some neoplasms including melanoma, leukemias, renal cell carcinoma (RCC), endometrial, ovarian, prostate, breast cancers, and some subtypes of sarcomas [[29], [30], [31], [32], [33]]. Molecular features of endoglin make it a reliable biomarker that may contribute malignant phenotypes in cancer to either an oncogenic or a non-oncogenic phenotype on the cell context. Although ENG, mainly is a co-receptor in the TGFβ pathway, plays significant roles in cell signaling followed by release from the cell membrane, as well. The presence of endoglin within the tumor microenvironment which expressed by neoplastic and non-neoplastic cells has opened a perspective for therapeutic purposes [28].

Evidence has shown that ENG suppresses the migration and invasion of prostate cancer cells and acts as a tumor-suppressor in prostate cancer [34]. Epigenetic suppression of ENG gene expression has been reported as a common event in some cancers such as esophageal squamous cell carcinoma and lung cancer, and a more aggressive phenotype results from this lack of expression [31,35].

In addition, other research has indicated that ENG plays an oncogenic role in some cases and causes tumor growth and development, and in some cases it will result in tumor suppression. These different roles depend on the cellular context [29]. Since in the present study, ENG downregulation apparently indicates the critical role of ENG as a tumor suppressor in the relevant tissue, and the reduction of its expression has led to the development of UCEC.

GNG4, as a member of the guanine nucleotide-binding protein complex [36] is a main component of heterotrimeric G protein. Evidently, GNG4 plays an essential role in the transmission of downstream signals of GPCRs to downstream pathways which leads to regulation of biological behaviors of normal and tumor cells [37]. The role of this protein in information transmission through several signaling pathways, including MAPK, PI3K, and Rho GEF pathways has been proven [38]. Recent research have indicated that GNG4 is up-regulated in several cancers including liver non-small-cell lung, bladder, and breast cancers, which is related to the poor prognosis [[39], [40], [41]]. Also, GNG4 can be introduced as a potential biomarker to predict immunotherapy response in bladder cancer [42]. Hyper methylation of GNG4 is reported in glioblastoma and bladder cancer [38,43]. Research as revealed that following the GNG4overexpression, proliferation of tumor is inhibited [38]. The focus of studies on the nervous system indicates that GNG4 is related to glioblastoma and cognitive decline [38,44]. Some studies have also suggested GNG4 as an unfavorable marker for rectal cancer and gallbladder cancer [40,41,45]. In previous studies, the tumor suppressor role of GNG4 has been mentioned in the core interaction network of colon cancer and it has been introduced as an important prognostic factor [46]. According to the tumor suppressor role of this protein, in the present study, GNG4 has downregulated, which will lead to tumor growth.

ECT2 is a proto-oncogene that encodes a guanine nucleotide exchange factor (EGF) for Rho GTPases [47]. Elevated ECT2 levels have been reported in various human tumors, including lung, pancreatic, liver cancers, and glioma [[48], [49], [50], [51]]. ECT2 as a GEF activates Rho family GTPases [52] that activates RhoA and serves as a signaling hub for different cellular processes such as cellular division, cell proliferation, and etc. The regulation of Ect2 activity remains inadequately comprehended [53]. Increasing evidence suggests that the dysregulation of Ect2 correlates with many malignancies, and the suppression of Ect2 has been shown to impede tumor growth, indicating that ECT2 functions as an oncoprotein and plays a crucial role in cancer progression [48,51,[54], [55], [56], [57]]. Also, ECT2 has been proposed as a diagnostic and prognostic biomarker in cervical and ovarian cancer [56]. Interestingly, the direct involvement of ECT2 in promoting DSB repair and essential maintenance of genome stability has been pointed out. In this case, ECT2 plays a role in HR and NHEJ repair pathways by facilitating BRCA1 and KU70 accumulation at DSB sites, and this mechanism is independent of ECT2's activity in canonical GEF pathway [58]. In various cancers, the expression of this protein starts in the S phase of the cell cycle and increases in the G2 and M phases [59]. It has been found that ECT2 crucially contributes in various cellular processes, including centromere maintenance, migration, cell adhesion, polarity, growth, and division [53,60,61]. Many evidences indicate that the irregular activity of ECT2 is involved in different cancer types and the reduction of ECT2 suppresses tumorigenesis [[62], [63], [64], [65]]. Therefore, studies showed oncogenic activity of ECT2 protein. High levels of DNA damage associated with replication stress are seen in tumor cells and it is necessary to repair these damages through HR to maintain cell survival [66,67]. Hence, it appears that ECT2 overexpression in cancerous cells is a way to repair DSB and overcome endogenous DNA damage, thereby maintaining their survival and promoting tumorigenesis. Therefore, from a therapeutic point of view, inhibition of ECT2 can lead to the possibility of increasing the therapeutic effectiveness during radiation therapy or chemotherapy. Knowing this function, ECT2 can be considered as one of the important genome caretaker. In our study, as expected, ECT2 upregulated which is another confirmation of its oncogenic role.

As mentioned in the results enriched pathways for these 304 DEGs were complement and coagulation cascades, cell adhesion molecules, human papillomavirus (HPV) infection, and ECM-receptor interaction. Today, the role of the ECM as a staple component of the cellular microenvironment in the development and regulation of normal tissues and homeostasis has been proven. Extensive structural remodeling and pathological dynamics in the ECM of the female reproductive tract contribute to various processes including cancer and metastasis [68]. Various glycoproteins, proteoglycans and other important cell signal transduction molecules that are components of the extracellular matrix also crucially contribute in the carcinogenesis [69]. Therefore, recognizing alterations in ECM components can be crucial in comprehending the mechanisms behind cancer growth and progression. The main components of the extracellular matrix can be used as potential biomarkers and new therapeutic strategies can be developed by studying their effect on surrounding tissues.

Human papilloma virus, as common sexually transmitted infection worldwide, is one of the most obvious risk factors for cancer. HPV is frequently implicated in the etiology of endometriosis, with its presence and genotypes initially examined in verified cases of the condition [70]. Research has shown that HPV E6 protein plays a vital role in inhibiting p53 protein function, and as a result, it leads to cell cycle inhibition and apoptotic progression [71]. Consistent with the results of studies that indicate that women with endometriosis develop hypercoagulability and this disorder is related to the inflammatory nature of the ectopic lesions [72,73], in our study one of enriched pathways for DEGs was complement and coagulation cascades. In fact, endometriosis is a chronic inflammatory disease, and the complement system is one of the important immune mechanisms involved in inflammation and autoimmunity in this disease. On the other hand, the close relationship between inflammatory conditions and coagulation has been recognized for years [[74], [75], [76]].

Since one of the vital processes in the migration of tumor cells is adhesion to the extracellular matrix [77], it is not far from expected that one of the important pathways in the development of many cancers, including UCEC, is pathways related to adhesion molecules. Several CAM genes involved in different aspects of cancer biology have been identified in previous studies [78] and by identifying more and more molecular events related to adhesion molecules, it will be possible to introduce confirmed molecules as promising biomarkers in the diagnosis, prognosis and treatment of cancer metastasis. Hence, disruption of CAM regulation is considered as an important characteristic of the most aggressive endometrial cancer [79]. Also, in confirming the role of adhesion molecules in UCEC, the correlation analysis of adhesion G protein-coupled receptors (GPCRs) and UCEC showed that some adhesion molecules up-regulated in UCEC tissues, which leads to adverse consequences and effects in patients [80].

In sum, in this study the key genes and signaling pathways that have an effect on the progression of endometriosis-related UCEC were identified using bioinformatics analyzes and surveys. The characteristics obtained in this study contribute to our further understanding of the basic processes involved in the development and recurrence of endometriosis-related UCEC. Although this research contains valuable information about potential targets in early diagnostic interventions and appropriate and timely treatment strategies for UCEC, validation of discovered genes and evaluation of identified signaling pathways in clinical samples seems necessary. However, experimental validation of bioinformatic data is always a necessity and requires careful examination of clinical samples. Briefly, the bioinformatics approach in this study resulted in the identification of important genes and pathways associated with UCEC progression following endometriosis. After experimental confirmation, three hub genes including ENG, GNG4 and ECT2 can be used in the early diagnosis of UCEC.

## 5 Conclusion

Nowadays, it is essential to identify the genes involved in cancer and the biological pathways that control tumor growth and development before conducting experimental tests using different bioinformatics methods. In this study using bioinformatics analysis 304 DEGs identified that contribute to the development of UCEC. Also PPI network analysis highlighted 78 hub genes and investigation of three selected genes including ENG, GNG4 and ECT2 revealed that may play critical regulatory roles in the cellular and molecular pathways including, human papillomavirus infection, complement and coagulation cascades, cell adhesion molecules, and ECM-receptor interaction. The identified hub genes, especially were found to be significantly dysregulated in UCEC tumor samples and may serve as promising diagnostic biomarkers. However, information obtained from the GO and KEGG pathway enrichment analyses offer critical insights into the principal genes and pathways influencing UCEC, in order to provide diagnostic biomarkers according to the results obtained from the present study, it seems necessary to conduct experimental tests and clinical samples.

## CRediT authorship contribution statement

**Mahsa Ejlalidiz:** Data curation, Formal analysis, Investigation, Methodology, Validation, Writing – review & editing. **Ameneh Mehri-Ghahfarrokhi:** Investigation, Methodology, Writing – review & editing. **Mohammadreza Saberiyan:** Conceptualization, Project administration, Supervision, Validation, Visualization, Writing – review & editing.

## Ethics approval and consent for participate

Not applicable.

## Funding

The authors declare that they have not received any funds.

## Declaration of competing interest

The authors declare that they have no known competing financial interests or personal relationships that could have appeared to influence the work reported in this paper.
