# Supplementary material for: Identification of hub genes and pathways in Uterine corpus endometrial carcinoma (UCEC): A comprehensive in silico study
Source: Biochem Biophys Rep. 2024 Nov 1;40:101860. doi: 10.1016/j.bbrep.2024.101860 (PMC11565547; doi:10.1016/j.bbrep.2024.101860)
Supplement: Multimedia component 1 [file mmc1.pdf]

Supplementary Tables

Supplementary Table 1. The common DEGs were screened according to adjusted P- values < 0.05 and logFC ≥ 1 and logFC ≤ −1.

CXCL12, OLFML1, COL6A2, TIMP2, NBL1, GSTM3, MAN1C1, MINOS1-NBL1///NBL1, TPM1, LAMA4, BAMBI, FHOD3, BGN, HTRA1, LTBP3, PTRF, RARRES2, PMP22, DKK 3.00, IGFBP6, PDGFRL, TIMP1, TAGLN, ENG, JAM3, AEBP1, DCN, FZD4, MYL9, ACTA2, JAM2, FMOD, WISP1, NID2, LAMB2, TNS1, LTBP2, LRP1, TPM2, SERPINF1, MGP, CTIF, PBXIP1, DPYSL3, THBS1, LBH, MIR6756///MCAM, MX1, DACT1, PLTP, IGFBP5, MMRN2, FXYD6, GRK5, MICAL2, CDKN1C, C1S, FMO1, MYLK, EGR1, KCNK3, OSMR, GLT8D2, CCL21, MN1, AQP1, PTGIS, ARHGAP22, SPSB1, FOSL2, NCF4, GGT5, CSDC2, C1QA, TPSB2///TPSAB1, ITM2A, MCAM, FXYD1, CNN1, KLF2, WISP2, LEFTY1///LEFTY2, VWF, APOL3, BMP6, C1R, LMOD1, KCND2, PLPP3, SYNPO, PBX3, SULF1, FHL2, IL4R, ENO2, ITGA8, THBS2, TSPAN7, JADE2, NUAK1, SLC4A3, PDE2A, CRYAB, RNASE6, ACACB, P3H2, SLCO2B1, PTGER3, C1QB, EPHX1, TPSAB1, FRZB, TSPAN4, PRELP, TPSB2, COLEC12, SLIT3, SCARB1, ACKR1, HLA-E, TRIL, GNG4, TCF21, NRP2, FZD7, C4B_2///C4B///C4A, TMEM176A, NCAM1, UCHL1, ELMO1, PAPSS2, GAS1, DOK5, TNXB///TNXA, LTC4S, QSOX1, PCOLCE2, TRPV2, HSPA12A, CLDN5, HOXC6, CPA3, KIAA1462, SEPT4, CPE, COL8A2, MFAP5, ITGA7, MYH11, SERPING1, TIMP3, NSG1, CLU, CCL15-CCL14///CCL14, INHBA, COL16A1, JUNB, EFEMP1, FOSB, RNASE1, NAV3, MMP23A///MMP23B, SIRPA, KCNMB1, TCEAL2, HLA-DPB1, TMEM176B, HSPB7, CDH3, C10orf10, BNC2, HLA-DPA1, THBD, SYNE3///LINC00341,WNT2B, BTG3, EHF, ITGB8, TPD52, LMNB1, NUS1P3, HOOK1, CDS1, SLC35A3, MAP2K6, ZBBX, MYO6, EXPH5, HPGD, PATJ, PRRG4, SLC39A8, LOC389906, IL20RA, RAD54B, MTHFD2L, RBBP8, PLS1, ARHGAP26, SMC2, REV3L, ANK3, TOM1L1, LOC401317///CREB5, LYPLA1, UGT8, MPPED2, CD44, OCLN, PAX8, PLA2G4A, CXADR, CWH43, CTAGE5, TXN, MFAP3L, FAM169A, SH3YL1, NUP62CL, TCF12, FZD5, SSX2IP, RBM47, ECT2, AMD1.00, CLMN, DUSP4, NCAPG2, ADAM28, SMC4, MPZL2, PLK4, LOC101929219///LOC100505650///C1orf186, ARSJ, LIMS4///LIMS1, GRAMD1C, LIMS4///LIMS3//LIMS1, CENPE, YME1L1, PLCB1, BTBD3, SLC15A2, HELLS, PERP, KIF18A, MECOM, FAM134B, ANXA3, PRR15L, SLC26A2, DNAJC10, SPAG1, ESRP1, LRRC1, GJA9-MYCBP///MYCBP, GALNT3, STIL, PAIP1, RAD51AP1, CCNE2, DNAJC15, AP1S2, SPA17, TRH, SHANK2, FOLH1B///FOLH1, DHFR, ASPM, PPA1, PTPN3, PRR5-ARHGAP8///ARHGAP8, ADGRG2, PIGR, SRD5A3, MAP7, PARPBP, CEACAM1, HMGB2, DEPDC1, NCAPG, BORA, CKAP2, DSP, HMMR, SLC7A1, CD24, EPCAM, MME, GRHL2, KIF11, TTK, TMEM30B, APOBEC3B, HEY2, MCOLN3, BRCA1, HSD17B2, PCNA, MAD2L1, PAX2, GPM6B, CDK1, CENPF, TNC

Supplementary Table 2. Network Specifications of common DEGs with Hub genes.

Network	Network diameter	Network density	Clustering coefficient	Characteristic path on length
234 nodes	9	0.037	0.338	3.480
78 nodes	5	0.117	0.493	2.454

Supplementary Table 3. 78 Hub genes from the PPI network of common DEGs (number of nodes: 78, clustering coefficient: 0.493, network centralization: 0.253).

COL6A2, IGFBP5, BGN, CCN4, SERPINF1, TPM1, MYLK, TXN, CNN1, TIMP3, TAGLN, MYL9, GNG4, MYH11, ENG, MCAM, COL16A1, CRYAB, THBS2, PRELP, ANK3, PLTP, PTGER3, ITGA7, NCAM1, AP1S2, HLA-E, HLA-DPA1, EGR1, PAX8, SULF1, KIF11, LMNB1, MME, KIF18A, PATJ, SIRPA, CENPE, PLK4, PAX2, HPGD, ENO2, PLA2G4A, KLF2, CENPF, SCARB1, DHFR, BRCA1, C1QA, CCL21, JAM3, HTRA1, PLCB1, VWF, CLDN5, CXCL12, ECT2, C1QB, IL4R, CD44, HMMR, CLU, ACTA2, DCN, CDK1, TIMP1, ESRP1, FMOD, THBS1, PCOLCE2, BMP6, MGP, FZD7, THBD, OCLN, DSP, FZD4, EPCAM
